# Different *Drosophila* cell types exhibit differences in mitotic centrosome assembly dynamics

**DOI:** 10.1016/j.cub.2015.05.061

**Published:** 2015-08-03

**Authors:** Paul T. Conduit, Jordan W. Raff

**Affiliations:** 1 Department of Zoology, University of Cambridge, Downing Street, Cambridge, CB2 3EJ, UK; 2 Sir William Dunn School of Pathology, University of Oxford, South Parks Road, Oxford OX1 3RE, UK

## Abstract

Centrosomes are major microtubule organising centres comprising a pair of centrioles surrounded by pericentriolar material (PCM). The PCM expands dramatically as cells enter mitosis, and we previously showed that two key PCM components, Centrosomin (Cnn) and Spd-2, cooperate to form a scaffold structure around the centrioles that recruits the mitotic PCM in *Drosophila*; the SPD-5 and SPD-2 proteins appear to play a similar function in *C. elegans*[1–3]. In fly syncytial embryos, Cnn and Spd-2 are initially recruited into a central region of the PCM and then flux outwards [4–6]. This centrosomal flux is potentially important, but it has so far not been reported in any other cell type. Here we examine the dynamic behaviour of Cnn and Spd-2 in *Drosophila* larval brain cells. Spd-2 fluxes outwards from the centrioles in both brains and embryos in a microtubule-independent manner. In contrast, although Cnn is initially incorporated into the region of the PCM occupied by Spd-2 in both brains and embryos, Cnn fluxes outwards along microtubules in embryos, but not in brain cells, where it remains concentrated around the centrosomal Spd-2. Thus, the microtubule-independent centrosomal-flux of Spd-2 occurs in multiple fly cell types, while the microtubule-dependent outward flux of Cnn appears to be restricted to the syncytial embryo.

## Main Text

**Figure 1:**
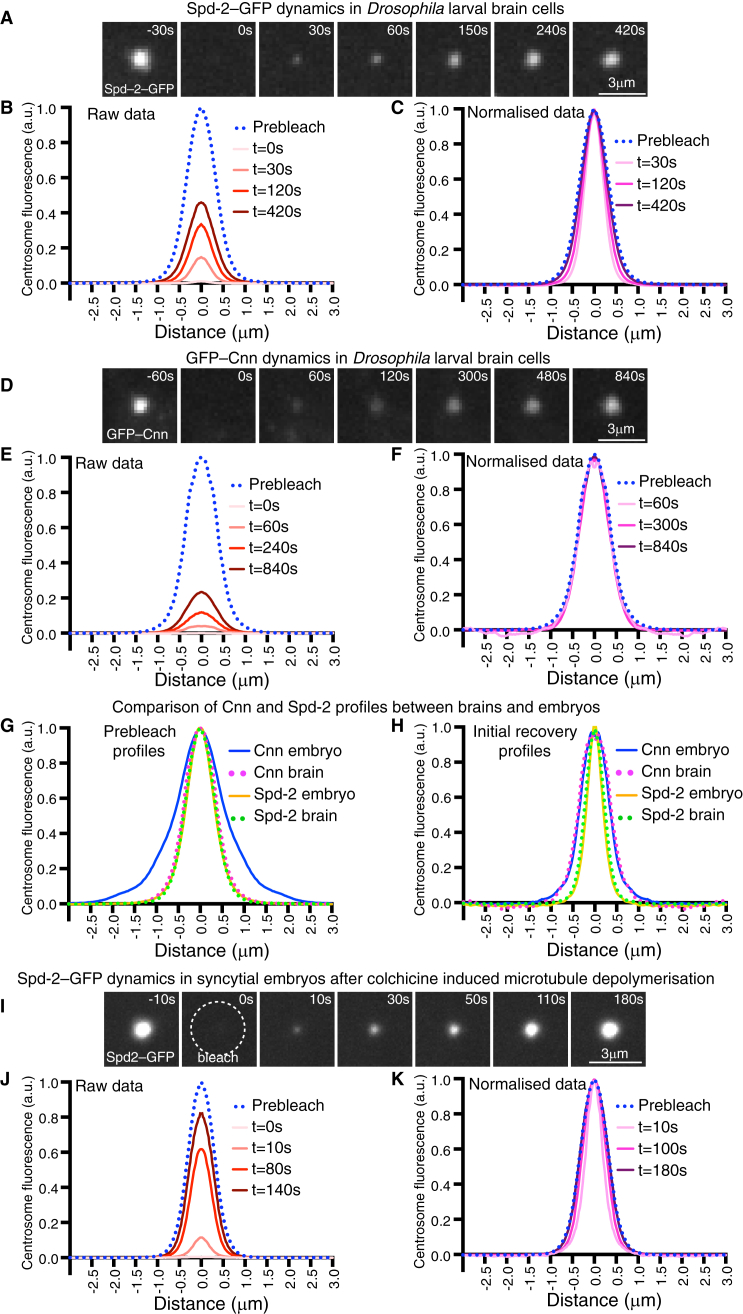
A comparison of the dynamic behaviour of Spd-2–GFP and GFP–Cnn in *Drosophila* larval brain cells and syncytial embryos. (A–F) Images (A,D) and graphs (B,C,E,F) show the dynamic behaviour of Spd-2–GFP (A–C) or GFP–Cnn (D–F) in *Drosophila* larval brain cells lacking endogenous Spd-2 or Cnn, respectively. Time before and after photobleaching (t = 0) is indicated. The graphs show the average fluorescence intensity profiles at selected time-points after photobleaching: (B) and (E) show the prebleached profiles (*dotted blue lines*) and successive ‘raw’ recovery profiles (*various shades of red*); (C) and (F) show the prebleached profiles and successive average normalized recovery profiles (*various shades of pink/purple*, normalized so that their peak intensity is equal to the peak intensity of the pre-bleached profile). The normalized recovery profiles of DSpd-2–GFP are initially narrower than the prebleached profile and become broader over time (p ≤ 0.001, F-test) (C); the normalized recovery profiles of GFP-Cnn are similar to the prebleached profile, and do not become broader over time (p = 0.18, F-test) (F). (G,H) Graphs compare the shapes of different profiles as indicated. (I–K) Images (I) and graphs (J,K) show the dynamic behaviour of Spd-2-GFP in *Drosophila* embryos that have been injected with colchicine; time before and after photobleaching (t = 0) is indicated. The graphs show the raw (J) and normalized (K) recovery profiles, as in B,C,E,F. The normalized recovery curves are initially narrower than the prebleached profile and spread outward over time (p < 0.0001, F-test).

We analysed the dynamic behaviour of Spd-2–GFP or GFP–Cnn at centrosomes in mitotic *Drosophila* larval brain cells using fluorescence recovery after photobleaching (FRAP). Both Spd-2–GFP and GFP–Cnn fluorescence recovered at centrosomes after photobleaching the centrosomal GFP signal, although Spd-2–GFP recovered faster than GFP–Cnn and both recovered more slowly than at embryonic centrosomes (Figure 1A–F; Figure S1A,B in the Supplemental Information) [6]. Normalising the recovery profiles allowed us to compare their shapes and revealed that, as in embryos, Spd-2–GFP fluorescence initially recovered only in the central region of the PCM and then spread outwards (Figure 1C), strongly suggesting that Spd-2–GFP molecules flux outwards from the centrioles in brain cells (Figure S1). Surprisingly, and in contrast to the situation in syncytial embryos [4,6], the GFP–Cnn fluorescence recovery profiles in brain cells were very similar in shape to the pre-bleached profile and did not spread outwards over time (Figure 1F).

In order to understand the difference between the distribution and dynamics of Cnn in brain cells and syncytial embryos, we compared the shapes of the pre-bleached and initial recovery profiles between the two cell types. Both the pre-bleached and initial-recovery profiles of Spd-2–GFP were very similar between embryos and brain cells (Figure 1G,H). In contrast, while the initial recovery profile of GFP–Cnn was very similar between embryos and brain cells (Figure 1H), the pre-bleached profile of GFP–Cnn was far more spread out in embryos (Figure 1G). This difference is likely due to the strong microtubule-dependent forces that move Cnn outwards in syncytial embryos, generating the phenomenon of ‘centrosomal flaring’ [5,7,8], that appear to be largely absent in brain cells (Figure S2A).

Interestingly, Spd-2–GFP does not appear to flare extensively in either embryos or brain cells (Figure S2A). We wondered, therefore, whether the centrosomal-flux of Spd-2–GFP occurs independently of microtubules. To test this, we examined the dynamic behaviour of Spd-2–GFP at centrosomes in syncytial embryos injected with the microtubule depolymerising drug colchicine. Remarkably, the dynamic behaviour of Spd-2–GFP was unperturbed: it continued to flux outwards from the centrioles at normal rates (Figure 1I–K, S2B), and, in contrast to GFP–Cnn [5], its levels at centrosomes remained roughly constant (Figure S2C).

Together with our previous findings, these data suggest that there are two phases to the expansion of the mitotic PCM in flies. In the first phase, which occurs in both embryos and brain cells, Spd-2 is incorporated around the wall of the mother centriole and then fluxes outwards in a microtubule independent manner. Spd-2 helps recruit other proteins into the PCM, but in the absence of Cnn it rapidly dissipates and cannot accumulate [6]. When Cnn is present, however, Spd-2 helps recruit it into the PCM where Cnn becomes phosphorylated by Polo and so assembles a multimeric Cnn scaffold [5]. This scaffold supports the outward expansion of Spd-2 and so expanded PCM recruitment [6]. Importantly, the expansion of Spd-2 in turn allows Cnn to be incorporated over a larger area, thus potentially establishing a positive feedback loop that ensures robust PCM assembly. In the second phase, which occurs in syncytial embryos but is largely absent in brain cells, the Cnn scaffold fluxes outwards along centrosomal microtubules, allowing the mitotic PCM to spread even further away from the centrioles.

Thus, Spd-2 in flies exhibits a genuine centrosomal flux that is microtubule-independent, while Cnn can build a supporting scaffold around the centrosomal Spd-2 without fluxing outwards; the microtubule-dependent outward flux of Cnn is only generated in specific cell types. Interestingly, SPD-5, which appears to perform a similar phospho-dependent scaffolding role to Cnn in worm embryos [9], does not flux outwards (see accompanying correspondence from Laos *et al.*). Clearly it will be interesting to determine whether Spd-2 homologues exhibit centrosomal-flux in other species.

## Supplemental Information

Document S1. Two Supplemental figures and experimental procedures
