# Supplementary material for: Different Drosophila cell types exhibit differences in mitotic centrosome assembly dynamics
Source: Curr Biol. 2015 Aug 3;25(15):R650–1. doi: 10.1016/j.cub.2015.05.061 (PMC4533225; doi:10.1016/j.cub.2015.05.061)
Supplement: Document S1. Two Supplemental figures and experimental procedures [file mmc1.pdf]

Supplementary Information

Different *Drosophila* cell types exhibit important differences in mitotic centrosome assembly dynamics

Paul T. Conduit and Jordan W. Raff

Inventory of supplemental information

Figure S1. A detailed analysis of Spd-2-GFP centrosomal-flux.

Figure S2. The outward flux of Spd-2-GFP is independent of microtubules.

Supplementary Experimental Procedures

Figure S1

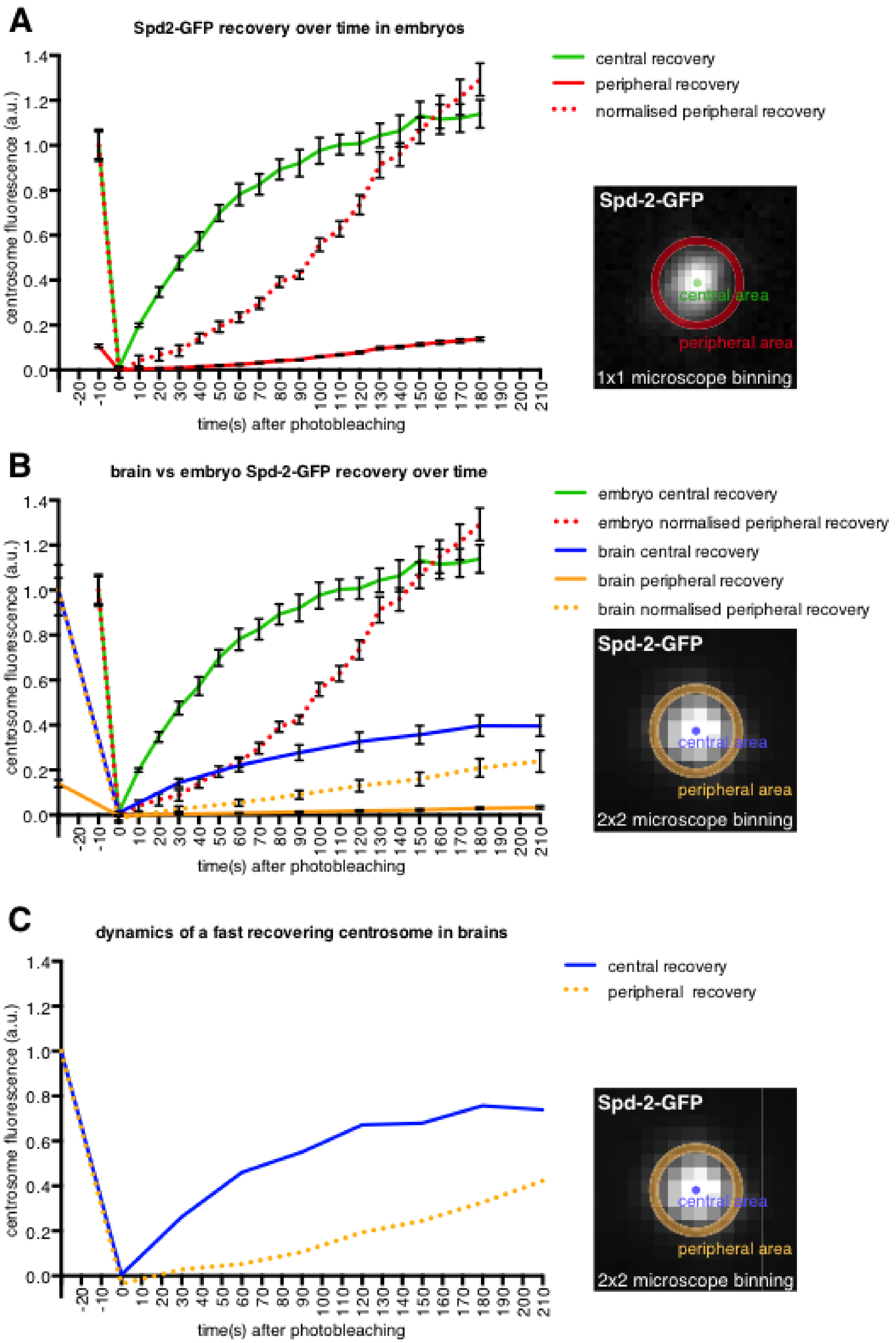
A
Spd2-GFP recovery over time in embryos
centrosome fluorescence (a.u.)
time(s) after photobleaching
central recovery
peripheral recovery
normalised peripheral recovery
Spd-2-GFP
central area
peripheral area
1x1 microscope binning
B
brain vs embryo Spd-2-GFP recovery over time
centrosome fluorescence (a.u.)
time(s) after photobleaching
embryo central recovery
embryo normalised peripheral recovery
brain central recovery
brain peripheral recovery
brain normalised peripheral recovery
Spd-2-GFP
central area
peripheral area
2x2 microscope binning
C
dynamics of a fast recovering centrosome in brains
centrosome fluorescence (a.u.)
time(s) after photobleaching
central recovery
peripheral recovery
Spd-2-GFP
central area
peripheral area
2x2 microscope binning

Figure S1. A detailed analysis of Spd-2–GFP centrosomal-flux. (A) In embryos, the recovery kinetics of Spd-2-GFP in the centre of the PCM display a typical logarithmic-shaped FRAP recovery curve (*green line*), while the recovery kinetics of Spd-2–GFP in the periphery of the PCM are unusual, with a slow initial rate that speeds up over time (*red* and *red dotted lines*) (as shown previously [S1]; data shown here is reproduced from this reference). These observations support the interpretation that Spd-2–GFP molecules spread outwards through the PCM, as in such a model one would expect that, while the fluorescence in the centre of the PCM recovers, the number of fluorescent molecules moving from the centre to the periphery per unit time would gradually increase over time (as discussed previously [S1]). (B) In brains, this analysis is complicated by the slow rate of recovery of Spd-2–GFP (compare *blue, orange* and *orange dotted lines* obtained in brains to the *green* and *red dotted lines* obtained in embryos), and because Spd-2–GFP fluorescence only recovers to ~40% of pre-bleached levels. Nevertheless, while the shape of the central recovery curve is clearly logarithmic (*blue line*), the shape of the peripheral recovery curve is not (*dotted orange line*). (C) This point is more clearly illustrated in this graph showing the central (*blue line*) and peripheral (*orange dotted line*) recovery dynamics at a single centrosome that happened to display unusually fast and high recovery rates. Here, the recovery in the periphery of the PCM starts slowly and speeds up over time, as observed in the embryo.

Figure S2

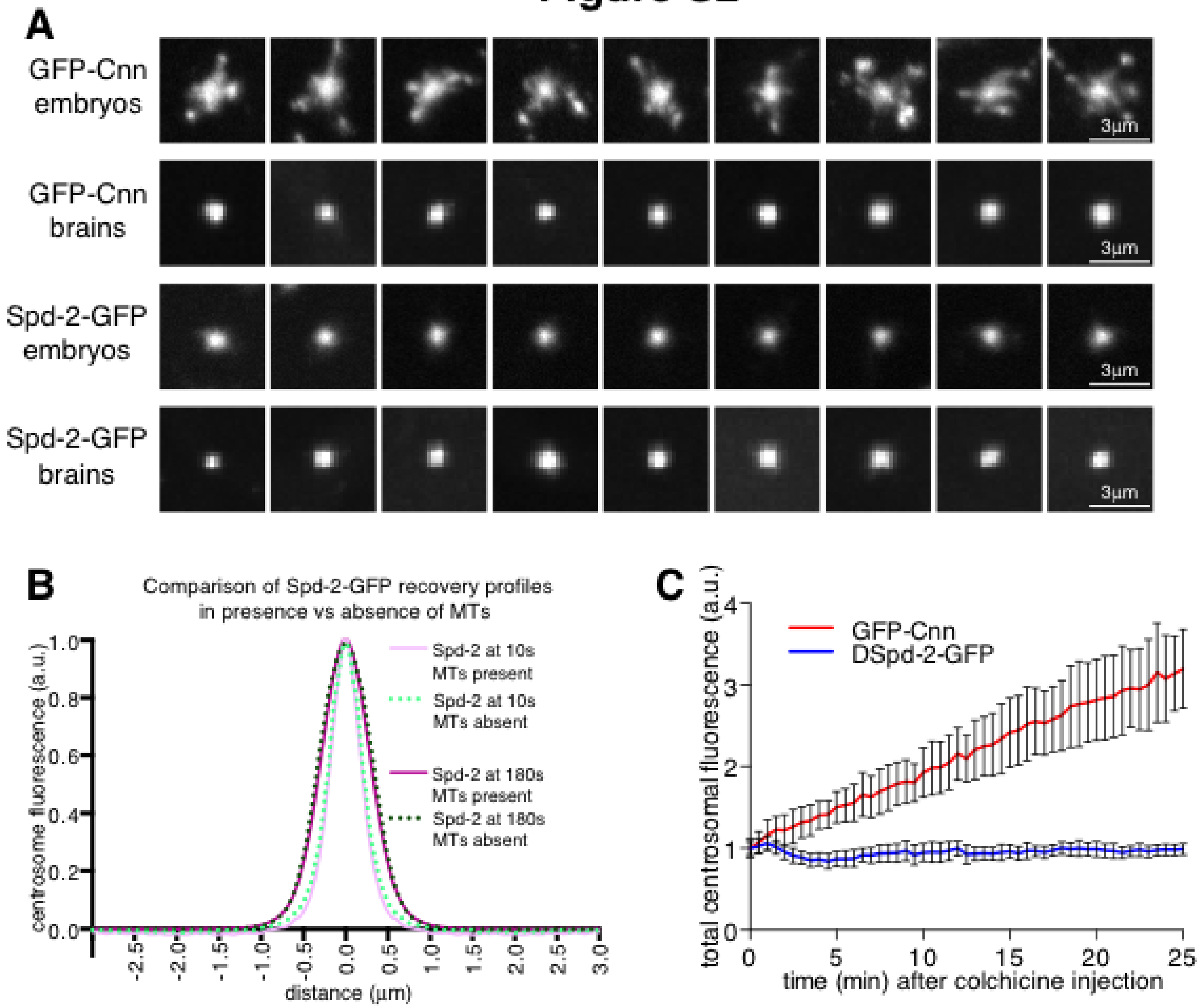
A
GFP-Cnn embryos
GFP-Cnn brains
Spd-2-GFP embryos
Spd-2-GFP brains
3µm
B
Comparison of Spd-2-GFP recovery profiles in presence vs absence of MTs
centrosome fluorescence (a.u.)
distance (µm)
Spd-2 at 10s MTs present
Spd-2 at 10s MTs absent
Spd-2 at 180s MTs present
Spd-2 at 180s MTs absent
C
total centrosomal fluorescence (a.u.)
time (min) after colchicine injection
GFP-Cnn
DSpd-2-GFP

Figure S2. The outward flux of Spd-2–GFP is independent of microtubules. (A) Images show randomly selected mitotic centrosomes from either embryos (rows 1 and 3) or brains (rows 2 and 4) expressing GFP–Cnn (rows 1 and 2) or Spd-2–GFP (rows 3 and 4). Note how in embryos GFP–Cnn stretches outwards and exhibits ‘centrosomal flaring’, which is known to be due to microtubule dependent forces [S2]. (B) Graph compares the average normalized initial recovery profiles (10s after photobleaching) and the average normalized profiles 180s after photobleaching of Spd-2–GFP in either the presence or absence of microtubules, as indicated; the absence of microtubules does not dramatically change the distribution of the profiles at each timepoint. (C) Graph compares the total fluorescence intensity of either Spd-2–GFP (blue line) or GFP–Cnn (red line) at centrosomes after the injection of colchicine. Error bars show standard error of the mean. Note how Spd-2–GFP levels stay roughly constant, showing that the Spd-2 molecules incorporating into the centre of the centrosome must still eventually leave the centrosome even in the absence of microtubules. In contrast, the Cnn molecules that incorporate into the centrosome no longer leave the centrosome in the absence of microtubules, so Cnn levels steadily increase over time.

Supplementary Experimental Procedures

Fly lines

1 copy of GFP–Cnn or 2 copies of Spd-2–GFP were expressed transgenically under the control of the pUbq promoter in a cnn^{f04547}/cnn^{HK21} or $spd\text{-}2^{z35711}/spd2^{df}$ genetic background, respectively. Both constructs rescue their respective mutant phenotypes (data not shown and [S3]) Similar to embryos

[S1], in brains GFP–Cnn is expressed at slightly higher levels than endogenous Cnn and Spd-2–GFP is expressed at slightly lower levels than endogenous Spd-2 (data not shown).

Larval brain preparation

Brains were dissected from 3rd instar larvae and placed in a 10μl drop of Schneider's medium supplemented with BSA and penicillum/streptavadin (Sigma) towards the edge of a 35mm MatTek glass bottom dish. A round coverslip was carefully placed on top in order to semi-squash the brain and extract cells from the edge of the brain. Mitotic Neuroblast cells (containing two mature centrosomes) that were either in prophase (pre-nuclear envelope breakdown) or pro-metaphase (post-nuclear envelope breakdown) at the edge of the brain close to the coverglass were selected for imaging. For Spd-2–GFP we bleached 10 centrosomes in prophase cells and 8 centrosomes in pro-metaphase cells; for GFP–Cnn we bleached 11 centrosomes in prophase cells and 2 centrosomes in pro-metaphase cells.

Imaging

Imaging was carried out on a Perkin Elmer Spinning Disk confocal system running Volocity software mounted on a Zeiss Axiovert microscope using a 60x/1.4 NA oil objective. Images shown are maximum intensity projections of several z-slices taken 0.5μm apart. Photobleaching of individual centrosomes was carried out using a focussed 440nm laser. Brain imaging was carried out in 2x2 binning mode due to low signal to noise compared to embryos. ImageJ was used to calculate the radial profiles of centrosomes at each timepoint. Images were scaled up either 5 times (1x1 bin embryo images) or 10 times (2x2 bin brain images) and the centre of mass of the centrosome was

calculated using the "analyse particles" function. Concentric rings spaced at 0.028μm (embryos) or 0.022μm (brains) and spanning across 3.02μm were centred and the average fluorescence around each ring was measured. For raw profiles, the data from several centrosomes at each timepoint was first averaged, and then the average cytosolic signal was subtracted from the average profiles. The average profiles were then normalized relative to the prebleached profile and mirrored to show a full symmetric centrosomal profile. For normalized profiles, each centrosomal profile was processed individually by subtracting the local cytosolic signal and normalising to a peak value of 1. Each profile was then mirrored to show a full symmetric centrosomal profile and the average of these normalized profiles was displayed in the graphs. To compare the normalized distribution at each timepoint, a guassian curve was fitted to each normalized centrosomal profile at each timepoint in GraphPad Prism and the standard deviations of these curves were compared using an F-test.

For analysing Spd-2–GFP in embryos lacking microtubules, colchicine was microinjected at a concentration of 1mM. The dynamics of centrosomal Spd-2–GFP under these conditions was analysed as described above; the embryos were in S-phase, which in these rapidly cycling embryos is when the centrosomes are in the process of mitotic maturation. To examine the change in the total amount of centrosomal Spd-2–GFP over time, the total Spd-2–GFP fluorescence signal at centrosomes was calculated at each timepoint using Volocity software. The local cytosolic fluorescence was subtracted and the data was normalized so the initial values were equal to 1. This data was compared to GFP–Cnn data obtained previously [S4].

Central versus peripheral recovery kinetics analysis

The central and peripheral PCM measurements were taken from the radial profile data described above. The average cytosolic signal at each timepoint was first subtracted from the profiles. Central and peripheral PCM measurements for each centrosome were calculated by averaging the fluorescence intensity of the 6 measurements taken between 0μm and 0.1096μm from the centre of each centrosome (as defined by the centre of mass of the fluorescent signal), or by averaging the fluorescence intensity of the 6 measurements taken between 0.6357μm and 0.7453μm from the centre of each centrosome. The average value of the 18 centrosomes analysed was calculated for each timepoint and the data was normalised so that the prebleached value of the central measurement equalled 1. To more easily compare the shapes of the central and peripheral recovery curves we also plotted a peripheral recovery curve that had been normalized so that its prebleached value equalled 1.

Supplemental References

S1. Conduit, P. T., Richens, J. H., Wainman, A., Holder, J., Vicente, C. C., Pratt, M. B., Dix, C. I., Novak, Z. A., Dobbie, I. M., Schermelleh, L., et al. (2014). A molecular mechanism of mitotic centrosome assembly in Drosophila. Elife http://dx.doi.org/10.7554/eLife.03399.

S2. Megraw, T.L., Kilaru, S., Turner, F.R., and Kaufnam, T.C. (2002). The centrosome is a dynamic structure that ejects PCM flares. J. Cell Sci. *115,* 4704-4718.

S3. Dix, C.I, and Raff, J.W. (2007) Drosophila Spd-2 recruits PCM to the sperm centriole, but is dispensable for centrosome duplication. Curr. Biol. *17* 1759-1764.

S4. Conduit, P.T, Feng, Z., Richens, J.H., Baumbach, J., Wainman, A., Bakshi, S.D., Dobbelaere, J., Johnson, S., Lea, S.M., and Raff, J.W. (2014) The centrosome-specific phosphorylation of Cnn by Polo/Plk1 drives Cnn scaffold assembly and centrosome maturation. Dev. Cell *28* 659-669.
